# The value of third-generation sequencing for neonatal screening of thalassemia in the Yulin region of Southern China

**DOI:** 10.3389/fgene.2026.1843092

**Published:** 2026-06-26

**Authors:** Sisi Ning, Shibin He, Guanghong Wei, Yi Liang, Yuling Xie, Jing Zhou, Xiafei Liang, Hui Liu, Aiping Mao, Yunrong Qin

**Affiliations:** 1 Department of Clinical Laboratory, Yulin Women and Children Health Care Hospital, Yulin, China; 2 Department of Research and Development, Berry Genomics Corporation, Beijing, China

**Keywords:** neonatal screening, precision prevention and control, rare variant, thalassemia, third-generation sequencing

## Abstract

**Background:**

Thalassemia is one of the most prevalent and severe monogenic disorders worldwide. In China, a three-tiered prevention and control strategy has been established for this disease, comprising preconception carrier screening, prenatal diagnosis, and neonatal screening. However, the implementation of neonatal thalassemia screening remains far behind the first two tiers, and the effectiveness of current screening strategies warrants systematic evaluation.

**Methods:**

To address this gap, we conducted a prospective neonatal screening for 800 newborns from the Yulin region by directly using third-generation sequencing (TGS), with the results rigorously compared to those obtained through conventional strategies.

**Results:**

The overall thalassemia carrier rate was 23.38%, with 14.75% for α-thalassemia, 6.50%for β-thalassemia, and 2.13% for combined α-/β-thalassemia, respectively. A total of 25 distinct variants were identified across the globin gene clusters, including 13 previously reported common variants in the Chinese population and 12 rare variants, underscoring substantial genetic heterogeneity and a considerable disease burden in this region. Comparative analysis revealed that hematological testing failed to detect 46 carriers, and 14 cases showed discordant genotypes between conventional genetic assays and TGS with all discordant variants validated by Sanger sequencing or multiple ligation probe amplification technology. Collectively, TGS increased the detection rate by 10.27%.

**Conclusion:**

These results demonstrate the comprehensive genomic landscape of thalassemia in the Yulin region. Furthermore, these findings provide evidence for the advantages of TGS over conventional strategies in neonatal thalassemia screening, thereby offering insights for the paradigm toward precision prevention and control of thalassemia, especially in high-prevalence regions.

## Introduction

Thalassemia is a hereditary monogenic disorder characterized by reduced or absent globin chain synthesis due to congenital defects in the globin genes (Higgs et al., 2012). In China, thalassemia is most prevalent in southern provinces, such as Guangxi, Guangdong, Yunnan, Guizhou, and Hainan (Wang et al., 2023). It is estimated that approximately 30 million individuals carrying thalassemia variants and 300,000 requiring medical intervention for thalassemia intermedia or major, raising a significant public health issue (Weatherall, 2001; Zeng and Huang, 1987; Cai et al., 2025; Foundation BAMC, 2020). To prevent and control thalassemia, China has established a three-tiered prevention strategy in high-prevalence areas, encompassing preconception carrier screening to identify couples at risk of conceiving offspring with moderate and severe thalassemia, prenatal diagnosis for high-risk pregnancies, and neonatal screening enabling early identification of affected neonates prior to symptom onset. Neonatal thalassemia screening offers several advantages for thalassemia prevention and control. First, it facilitates prompt clinical management and individualized treatment for affected infants. Second, it provides longitudinal evidence to guide future reproductive risk assessment and partner testing strategies. Therefore, comprehensive and accurate neonatal screening is crucial for effective thalassemia management at the public health level. However, the implementation of neonatal thalassemia screening lags behind that of carrier screening and prenatal diagnosis.

The Yulin Region comprises seven counties, spans 12,800 km^2^, and serves a population of 7.44 million, making it the second most populous city in Guangxi Zhuang Autonomous Region, China. As part of a historically high-prevalence region, Yulin faces substantial challenges in the prevention and control of thalassemia (He et al., 2018). Prior epidemiological investigations in this region predominantly focus on preconception and prenatal population (He et al., 2018; Li et al., 2020), leaving a pronounced paucity of population-representative epidemiological data, variant spectrum, and screening performance among newborns. To bridge this gap, we enrolled 800 consecutive newborns delivered at the Yulin Women and Children Health Care Hospital for thalassemia screening. All participants underwent parallel, blinded screening using third-generation sequencing (TGS) and conventional screening strategies simultaneously. The primary aims of the study included: (1) establishment of baseline carrier rate of thalassemia in this understudied population, thereby supporting further clinical management; (2) rigorous evaluation of the effectiveness of TGS technology in neonatal thalassemia screening over conventional screening strategies, providing new insights for precision prevention and control of thalassemia, especially in areas with high-burden of thalassemia.

## Materials and methods

### Study participants

During the period September to December 2023, all neonates born in the Yulin Women and Children Health Care Hospital who met the eligibility criteria were consecutively enrolled until the target sample size of 800 was reached. Inclusion criteria comprised no history of blood transfusion within 7 days after birth and parental household registration within the Yulin Region. Exclusion criteria were a diagnosis of severe hemolytic disease or congenital heart disease. The study protocol received approval from the Medical Ethics Committee of Yulin Women and Children Health Care Hospital. All parents or guardians had given their informed consent.

### Samples

Heel blood samples were collected from all subjects for hemoglobin electrophoresis and genetic analysis. Collect three to five drops of heel blood from the lateral ankle area of each newborn, which is 2–7 days old and has received adequate breastfeeding. The blood was spotted onto TF blood collection cards. Two cards were prepared per individual, each containing at least three blood spots (diameter >8 mm). After air-drying, the cards were packaged in separate sealed bags and stored at −20 °C.

### Dried blood spot (DBS) capillary hemoglobin electrophoresis

Hemoglobin separation and quantification of heel blood DBS was performed using the Sebia Capillarys 2 Neonat Fast™ instrument (Sebia Co., Paris, France). Samples with positive screening results or abnormal hemoglobin electrophoresis bands (peaks) underwent routine thalassemia genetic analysis.

### Genotypic analysis using conventional methods

A Lab-Aid 820 Nucleic Acid Extractor (Xiamen Zhishan Biotechnology, Xiamen, China) was used to extract genomic DNA from the DBS sample, which was then stored at −20 °C. The following genotyping techniques were used: gap-polymerase chain reaction (Gap-PCR) (Yisheng tang, Shenzhen, China) for four common deletional α-thalassemia (--^SEA^, --^THAI^, -α^3.7^, -α^4.2^), PCR-reverse dot blot (PCR-RDB) (Yaneng, Shenzhen, China) for three common non-deletional variants of α-thalassemia (Hb Constant Spring (CS): c.427T>C; Hb Quong Szec (QS): c.377T>C; Hb Westmead (WS): c.369C>G) and 17 common point mutations of β-thalassemia (βCD41/42(-CTTT): c.126_129delCTTT; βIVS-II-654(C>T): c.316–197C>T; βCD17(AAG>TAG): c.52A>T; β-28(A>G): c.-78A>G; β-29(A>G): c.-79A>G; β-30(T>C): c.-80T>C; β-32(C>A): c.-82C>A; βCD26(GAG>AAG): c.79G>A; βCD71/72(+A): c.216_217insA; βCD43(GAG>TAG): c.130G>T; βIVS-I-1(G>T): c.92 + 1G>T; βCD27/28(+C): c.84_85insC; βInt(T>G): c.2T>G; βCD31(-C): c.94delC; βCap+40–43(-AAAC): c.-11_-8delAAAC; βCD14/15(+G): c.45_46insG; βIVS-I-5(G>C): c.92 + 5G>C). These variants were grouped as common variants.

### Genotypic analysis using TGS

All DBS samples were subjected to single-molecule real-time (SMRT) technology-based TGS, as previously described (Ning, et al., 2023). First, multiplex long-range PCR was conducted on genomic DNA with optimized primers designed to cover known structural variants (SVs), single-nucleotide variants (SNVs), and insertions-deletions (Indels) in the *HBA1*, *HBA2*, and *HBB* genes, as well as HS-40 deletion. Samples were considered qualified if successful long-range PCR was achieved, as confirmed by the presence of visible HBA1/2 and HBB gene amplicons following agarose gel electrophoresis. Subsequently, amplified PCR fragments underwent end-repair and ligation of hairpin adaptors to the 5′ and 3′ ends, yielding individual dumbbell-shaped pre-libraries. Equal amounts of each pre-library were mixed with sequencing primers and DNA polymerase to construct the PacBio sequencing library. These primer-polymerase complexes were loaded onto a SMRT cell (Pacific Biosciences) for sequencing on a PacBio Sequel II system, which yielded 10–25 sub-reads per molecule. The sub-reads were processed to generate circular consensus sequencing (CCS) reads, which were subsequently aligned to the GRCh38 reference sequence. The mean sequencing depth across target regions was >500x. Variant calling was performed using FreeBayes software (version 1.3.4). For quality SNV/Indel calling, a pass was given when variant/wild-type reads were >20% and total CCS reads were >100; for quality SV calling, a pass was given when variant/wild-type reads were >5% and total CCS reads were >50. The variants were classified for pathogenicity according to established guidelines and hemoglobin variant databases (the HbVar, Ithanet, LOVD, and LOVD-China databases), and final phenotypes were assigned based on recognized genotype-phenotype associations.

### Validation of samples with inconsistent test results

For samples with inconsistent results from the two detection routes, the genotypes were verified by PCR-electrophoresis, multiplex ligation-dependent probe amplification (MLPA, MRC Holland, Amsterdam, Netherlands) or Sanger sequencing methods.

### Statistical analysis

SPSS25.0 software (IBM, Chicago, IL, United States) was used for data analysis. The chi-square test was used to evaluate the significance of the differences in genotype frequencies between sexes. A two-tails P-value of less than 0.05 was considered statistically significant.

## Results

### Carrier rates of thalassemia identified by TGS

**FIGURE 1:**
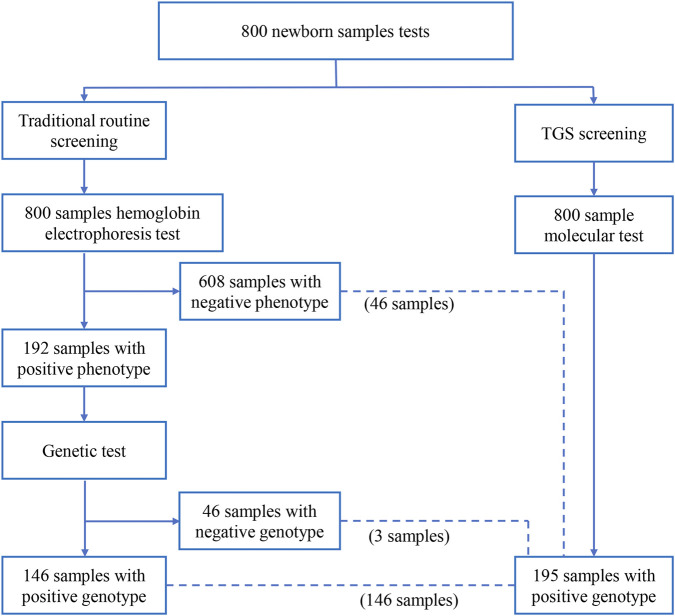
Comparison of newborns with thalassemia identified by using conventional screening workflows and TGS method.

A total of 800 eligible neonates including 378 females and 422 males were prospectively enrolled for thalassemia screening. All participants underwent parallel, blinded analysis using TGS and conventional screening workflows (Figure 1). TGS identified 187 carriers (23.38%) with thalassemia variants, comprising 118 (14.75%) with α-thalassemia variants, 52 (6.50%) with β-thalassemia variants, and 17 (2.13%) with both α- and β-thalassemia variants (Table 1). No statistically significant difference was observed in the overall carrier rate between sexes (P = 0.370). However, sex-stratified analyses revealed significant disparity in the carrier rate of α-thalassemia (P = 0.019). In addition, six carriers of α-triplication or quadruplication and two carriers of non-thalassemic hemoglobinopathies were detected.

**TABLE 1:** Results of neonatal thalassemia screening by TGS.

Gender	NO.	Total	Thalassemia carriers			Others	
			α-thalassemia	β-thalassemia	Combined α/β-thalassemia	α-structural	Hemoglobinopathies
Total	800	187 (23.38%)	118 (14.75%)	52 (6.50%)	17 (2.13%)	6 (0.75%)	2 (0.25%)
Female	378	83 (21.96%)	44 (11.64%)	29 (7.67%)	10 (2.65%)	2 (0.53%)	1 (0.26%)
Male	422	104 (24.64%)	74 (17.54%)	23 (5.45%)	7 (1.66%)	4 (0.95%)	1 (0.24%)

### Genetic landscape of thalassemia revealed by TGS

A total of 25 distinct variants across 225 mutant alleles were identified by TGS. Among these, 12 variant types accounted for 151 mutant alleles in the *HBA1*/2 gene locus, while 13 variant types accounted for 74 mutant alleles in the *HBB* gene locus (Table 2). Among α-globin gene variants, six common variants collectively represented 91.39% (138/151) of all mutant alleles, while the remaining 8.61% were from six rare variants including three structural variants (ααα^anti3.7^, ααα^anti4.2^, and αααα^anti3.7^) and three SNVs (*HBA2*:c.-15C>G, *HBA1*:c.84G>T, and *HBA2*:c.300 + 43G>A). Among β-globin gene variants, only seven common variants were detected; the remaining six rare variants collectively represented 36.49% of all mutant alleles, including *HBB*:c.316–179A>C, *HBB*:c.316–45G>C, *HBB*:c.315 + 180T>C, *HBB*:c.-100G>A, *HBB*:c.315 + 308delA, and *HBB*:c.315 + 5G>C, underscoring substantial diversity in this cohort. The hematological results of cases with rare variants were shown in Supplementary Table S1.

**TABLE 2:** Variants identified by TGS and conventional methods in neonates from the Yulin region.

Variants	Clinical significance^a^	Allele (n)	CR (%)	Detection range of routine methods
α-globin gene	​	**151**	**100.00**	​
--^SEA^	Pathogenic	60	39.74	Yes
-α^3.7^	Pathogenic	33	21.85	Yes
-α^4.2^	Pathogenic	21	13.91	Yes
*HBA2*:c.369C>G	Uncertain significance	12	7.95	Yes
*HBA2*:c.427T>C	Pathogenic	8	5.31	Yes
ααα^anti3.7^	Uncertain significance	5	3.31	No
*HBA2*:c.377T>C	Pathogenic	4	2.65	Yes
*HBA2*:c.-15C>G	Uncertain significance	3	1.99	No
*HBA1*:c.84G>T	Uncertain significance	2	1.32	No
*HBA2*:c.300 + 43G>A	Uncertain significance	1	0.66	No
ααα^anti4.2^	Uncertain significance	1	0.66	No
αααα^anti3.7^	Uncertain significance	1	0.66	No
β--globin gene	​	**74**	**100.00**	​
*HBB*:c.126_129delCTTT	Pathogenic/Likely pathogenic	23	31.08	Yes
*HBB*:c.316–179A>C	Uncertain significance	11	14.86	No
*HBB*:c.316–197C>T	Pathogenic/Likely pathogenic	7	9.46	Yes
*HBB*:c.316–45G>C	Uncertain significance	6	8.11	No
*HBB*:c.52A>T	Pathogenic/Likely pathogenic	6	8.11	Yes
*HBB*:c.-78A>G	Pathogenic/Likely pathogenic	5	6.76	Yes
*HBB*:c.315 + 180T>C	Uncertain significance	5	6.76	No
*HBB*:c.-100G>A	Pathogenic/Likely pathogenic	3	4.05	No
*HBB*:c.216_217insA	Pathogenic/Likely pathogenic	3	4.05	Yes
*HBB*: c. −79A>G	Pathogenic/Likely pathogenic	2	2.70	Yes
*HBB*:c.315 + 308delA	Uncertain significance	1	1.35	No
*HBB*:c.315 + 5G>C	Pathogenic/Likely pathogenic	1	1.35	No
*HBB*:c.79G>A	Pathogenic/Likely pathogenic	1	1.35	Yes
Total	​	**225**	**100.00**	**-**

^a^ data from thalassemia database, including ithanet, HbVar, and LOVD. CR, constitute ratio. Bold values indicate the thalassemia variant types identified in this study.

Among the 118 α-thalassemia carriers, twelve distinct genotypes were detected (Table 3). --^SEA^/αα (43.22%, 51/118), -α^3.7^/αα (22.03%, 26/118), and -α^4.2^/αα (13.56%, 16/118) were the top three common genotypes. According to the genotypes, 63 (53.39%) carriers were predicted to have α-thalassemia silent, 54 (45.76%) α-thalassemia minor, and one (0.85%) α-thalassemia intermedia. Among the 52 β-thalassemia carriers, fifteen genotypes were observed, all of which resulted from point mutations or small Indels (Table 3). The most frequent genotype was heterozygous *HBB*:c.126_129delCTTT (30.77%, 16/52). Phenotypic classification indicated that 48 (92.31%) carriers were heterozygotes consistent with β-thalassemia minor, while four (7.69%) carriers were compound heterozygous genotypes compatible with β-thalassemia intermedia. Among the 17 carriers with combined α-/β-thalassemia, fourteen distinct genotype combinations were detected (Table 3), of which the most common was the combination of--^SEA^/αα and *HBB*:c.126_129delCTTT heterozygote (17.65%).

**TABLE 3:** Genotypes of neonates identified by TGS.

Genotypes	All		Female		Male	
	NO.	CR (%)	NO.	CR (%)	NO.	CR (%)
α-thalassemia	**118**	100.00	**44**	100.00	**74**	100.00
--^SEA^/αα	51	43.22	21	43.73	30	40.54
-α^3.7^/αα	26	22.03	7	15.91	19	25.68
-α^4.2^/αα	16	13.56	7	15.91	9	12.16
*HBA2*:c.427T>C/+	7	5.93	3	6.82	4	5.41
*HBA2*:c.369C>G/+	7	5.93	3	6.82	4	5.41
*HBA2*:c.377T>C/+	4	3.39	1	2.27	3	4.05
*HBA2*:c.-15C>G,c.369C>G/+	2	1.66	0	0.00	2	2.70
--^SEA^/-α^3.7^	1	0.85	0	0.00	1	1.35
*HBA2*:c.300 + 43G>A/+	1	0.85	1	2.27	0	0.00
--^SEA^/ααα^anti3.7^	1	0.85	1	2.27	0	0.00
-α^3.7^/*HBA2*:c.-15C>G,c.369C>G	1	0.85	0	0.00	1	1.35
-α^4.2^/*HBA2*:c.369C>G	1	0.85	0	0.00	1	1.35
Hemoglobinopathies	**2**	**100.00**	**1**	**100.00**	**1**	**100.00**
*HBA1*:c.84G>T/+	2	100.00	1	100.00	1	100.00
α-triplication	**6**	**100.00**	**2**	**100.00**	**4**	**100.00**
ααα^anti3.7^/αα	4	66.67	2	100	2	50.00
ααα^anti4.2^/αα	1	16.67	0	0.00	1	25.00
αααα^anti3.7^/αα	1	16.67	0	0.00	1	25.00
α-/β-thalassemia	**17**	**100.00**	**10**	**100.00**	**7**	**100.00**
--^SEA^/αα & *HBB*:c.126_129delCTTT/+	3	17.65	1	10.00	2	28.57
-α^4.2^/αα & *HBB*:c.126_129delCTTT/+	2	11.76	2	20.00	0	0.00
-α^3.7^/αα & *HBB*:c.-100G>A/+	1	5.88	1	10.00	0	0.00
-α^3.7^/αα & *HBB*:c.316–179A>C/+	1	5.88	1	10.00	0	0.00
-α^3.7^/αα & *HBB*:c.316–45G>C/+	1	5.88	0	0.00	1	14.29
-α^4.2^/αα & *HBB*:c.-79A>G/+	1	5.88	1	10.00	0	0.00
-α^4.2^/αα & *HBB*:c.316–45G>C/+	1	5.88	1	10.00	0	0.00
--^SEA^/αα & *HBB*:c.52A>T/c.-100G>A	1	5.88	1	10.00	0	0.00
--^SEA^/αα & *HBB*:c.216_217insA/+	1	5.88	0	0.00	1	14.29
--^SEA^/αα & *HBB*:c.316–45G>C/+	1	5.88	1	10.00	0	0.00
*HBA2*:c.369C>G/+ & *HBB*:c.126_129delCTTT/+	1	5.88	1	10.00	0	0.00
*HBA2*:c.427T>C/+ & *HBB*:c.216_217insA/+	1	5.88	0	0.00	1	14.29
--^SEA^/-α^3.7^ & *HBB*:c.52A>T/+	1	5.88	0	0.00	1	14.29
-α^3.7^/αα & *HBB*:c.315 + 180T>C/+	1	5.88	0	0.00	1	14.29
β-thalassemia	**52**	100.00	**29**	100.00	**23**	100.00
*HBB*:c.126_129delCTTT/+	16	30.77	9	31.03	7	30.43
*HBB*:c.316–179A>C/+	8	15.38	4	13.79	4	17.39
*HBB*:c.316–197C>T/+	6	11.54	4	13.79	2	8.70
*HBB*:c.-78A>G/+	5	9.62	4	13.79	1	4.35
*HBB*:c.315 + 180T>C/+	4	7.69	2	6.90	2	8.70
*HBB*:c.316–45G>C/+	3	5.77	0	0.00	3	13.04
|*HBB*:c.52A>T/+	2	3.85	0	0.00	2	8.70
*HBB*:c.-100G>A/+	1	1.92	1	3.45	0	0.00
*HBB*:c.126_129delCTTT/c.79G>A	1	1.92	1	3.45	0	0.00
*HBB*:c.216_217insA/+	1	1.92	0	0.00	1	4.35
*HBB*:c.315 + 308delA/+	1	1.92	1	3.45	0	0.00
*HBB*:c.315 + 5G>C/+	1	1.92	1	3.45	0	0.00
*HBB*:c.316–197C>T/c.52A>T	1	1.92	0	0.00	1	4.35
*HBB*:c.316–179A>C/c.-79A>G	1	1.92	1	3.45	0	0.00
*HBB*:c.316–179A>C/c.52A>T	1	1.92	1	3.45	0	0.00
Total	**195**	​	**86**	​	**109**	​

CR, constitute ratio. Bold values indicate the thalassemia variant types identified in this study.

### Comparative performance of conventional screening strategies versus TGS

In the conventional screening workflow, neonates initially underwent hemoglobin electrophoresis: 608 cases yielded negative results while 192 showed positive results (Figure 1). Subsequent confirmatory genetic testing using Gap-PCR and PCR-RDB identified common variants in 146 of 192 cases with positive hematological results (Figure 1), eventually yielding an overall carrier rate of 18.25% (146/800). These included 109 α-thalassemia, 26 β-thalassemia, and 11 combined α/β-thalassemia carriers.

**FIGURE 2:**
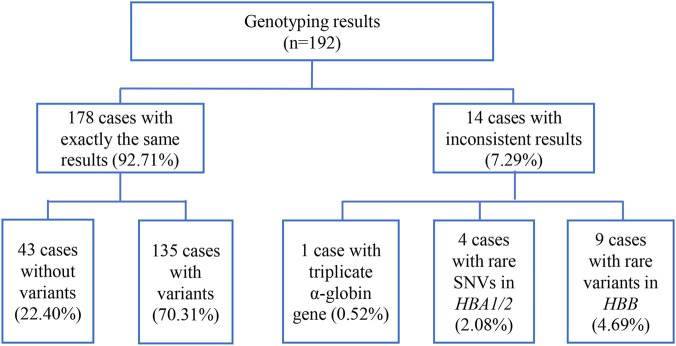
Genotype comparison between PCR-based genetic assays and TGS technology.

**FIGURE 3:**
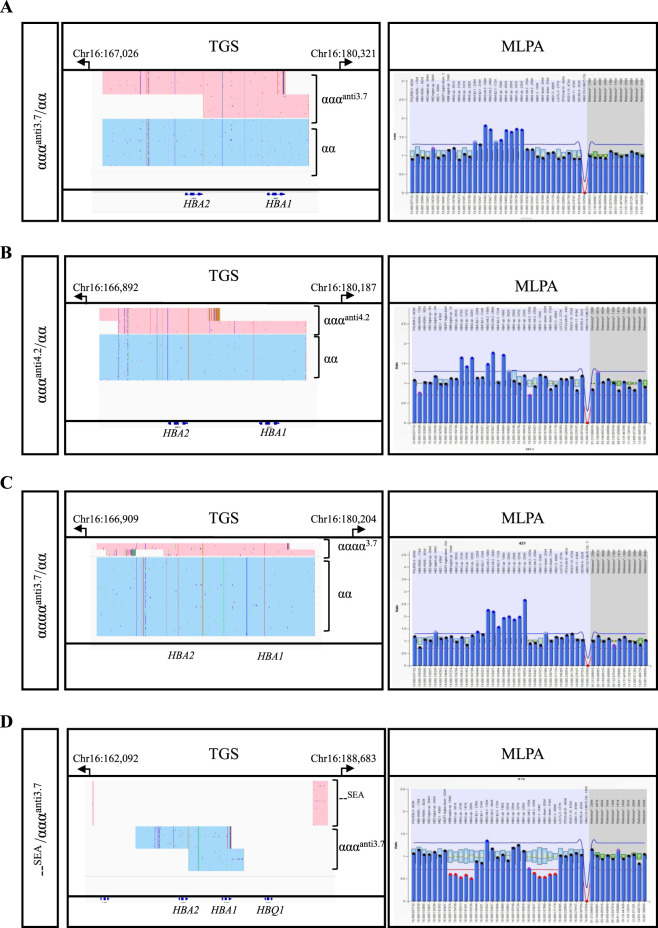
Visualization of rare *HBA1*/2 structural variants **(A)** ααα^anti3.7^/αα **(B)** ααα^anti4.2^/αα **(C)** αααα^anti3.7^/αα; and **(D)** --^SEA^/ααα^anti3.7^. Left panel, Integrative Genomics Viewer (IGV) plots of results of TGS. The blue and pink areas represent two chromosomes. Right panel, validation results obtained through MLPA.

**FIGURE 4:**
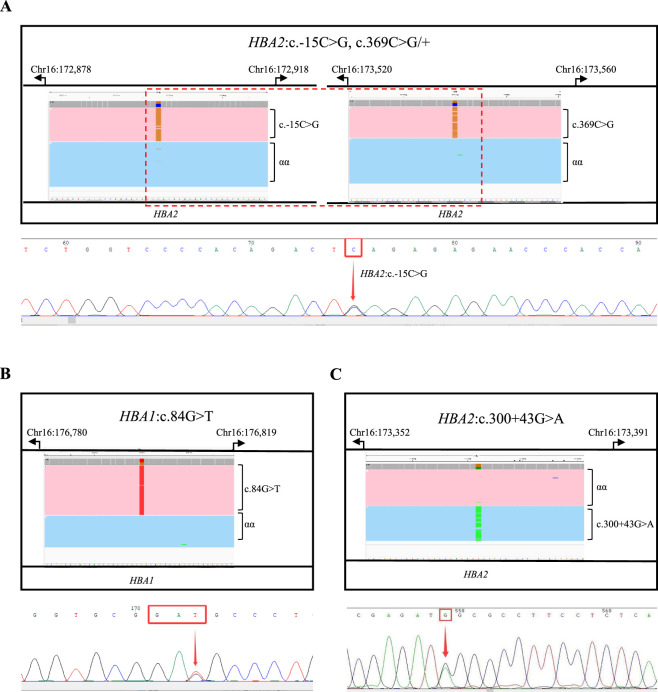
Visualization of rare *HBA1*/2 small variants **(A)** *HBA2*:c.-15C>G, c.369C>G heterozygote; **(B)** *HBA1*:c.84G>T heterozygote; and **(C)** *HBA2*:c.300+43G>A heterozygote. Upper panel, Integrative Genomics Viewer (IGV) plots of results of TGS. The blue and pink areas represent two chromosomes. Lower panel, validation results obtained through Sanger sequencing.

**FIGURE 5:**
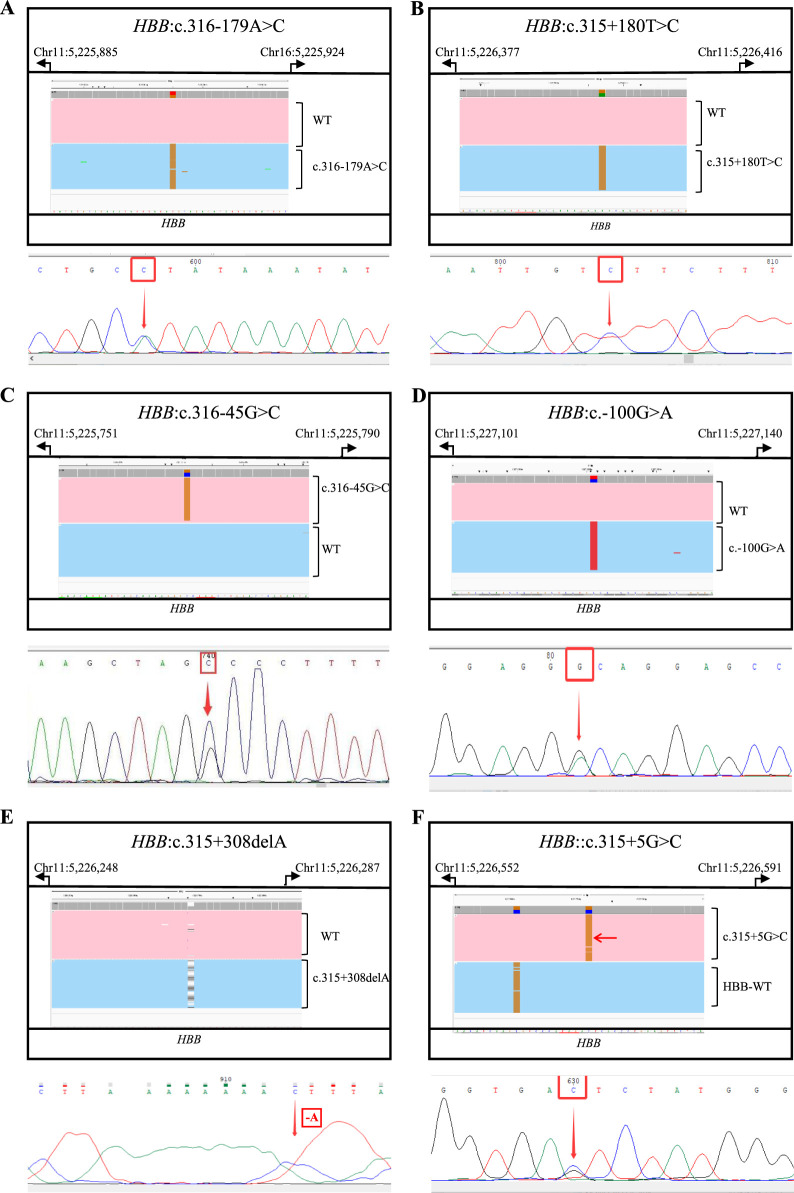
Visualization of rare *HBB* small variants **(A)** *HBB*:c.316–179A>C heterozygote **(B)** *HBB*:c.315 + 180T>C heterozygote **(C)** *HBB*:c.316–45G>C heterozygote **(D)** *HBB*:c.-100G>A heterozygote **(E)** *HBB*:c.315 + 308delA heterozygote; and **(F)** *HBB*:c.315 + 5G>C heterozygote. Upper panel, Integrative Genomics Viewer (IGV) plots of results of TGS. The blue and pink areas represent two chromosomes. Lower panel, validation results obtained through Sanger sequencing. WT: Wild type.

Rigorous comparison with TGS results revealed two critical limitations of the conventional screening strategies. First, TGS detected clinically relevant globin gene variants in 46 cases with negative hematological results, thereby increasing the detection rate by 23.96% (46/192) relative to hematological testing. Twenty two of them can be detected as carriers by PCR-based assays, while the remaining can be detected only by TGS (Table 4). Second, among the 192 cases with positive hematological results, 14 exhibited discordant genetic findings between conventional PCR-based assays and TGS. All discordances were caused by additional detection of rare variants by TGS, including five due to rare α-globin gene variants and nine due to rare β-globin gene variants (Figure 2; Table 5). Sanger sequencing and MLPA independently validated all discordant variants, confirming the accuracy of TGS. Representative long-read alignment for these variants were visualized in the Integrative Genome Viewer (Figures 3–5). Collectively, TGS detected 195 cases with globin gene variants, yielding an increment of 33.56% (49/146) over conventional screening workflow.

**TABLE 4:** Details of positive TGS detection results in the hemoglobin electrophoresis screening negative group.

Genotypes	NO.	Frequency (%)
*HBA1*/2 deletions	7	0.88
-α^4.2^/αα	6	0.75
-α^3.7^/αα	1	0.13
*HBA1*/2 SNV/InDels	**8**	**1.00**
*HBA2*:c.369C>G/+	6	0.75
*HBA2*:c.-15C>G,c.369C>G/+	1	0.13
*HBA1*:c.84G>T/+	1	0.13
*HBA1*/2 SV	**6**	**0.75**
ααα^anti3.7^/αα	4	0.50
ααα^anti4.2^/αα	1	0.13
αααα^anti3.7^/αα	1	0.13
*HBB* SNV/InDels	**25**	**3.13**
*HBB*:c.316–179A>C/+	7	0.88
*HBB*:c.315 + 180T>C/+	4	0.50
*HBB*:c.126_129delCTTT/+	3	0.38
*HBB*:c.316–45G>C/+	3	0.38
*HBB*:c.316–197C>T/+	2	0.25
*HBB*:c.-78A>G/+	1	0.13
*HBB*:c.-100G>A/+	1	0.13
*HBB*:c.216_217insA/+	1	0.13
*HBB*:c.315 + 308delA/+	1	0.13
*HBB*:c.315 + 5G>C/+	1	0.13
*HBB*:c.316–179A>C/c.-79A>G	1	0.13
Total	**46**	**5.75**

TGS, third-generation sequencing; SNV, single-nucleotide variant; InDels, insertion and deletions; SEA, south east asian. Bold values indicate the thalassemia variant types identified in this study.

**TABLE 5:** Thalassemia variants identified by TGS and conventional methods in the hemoglobin electrophoresis screening positive group.

Genotypes	TGS	PCR-based assays			Validation
	NO.	Accordance (NO.)	Discordance (NO.)	Genotype	Genotype
*HBA1/2* deletions	87	87	-	-	-
--^SEA^/αα	51	51	**-**	**-**	**-**
-α^3.7^/αα	25	25	**-**	**-**	**-**
-α^4.2^/αα	10	10	**-**	**-**	**-**
--^SEA^/-α^3.7^	1	1	**-**	**-**	**-**
*HBA1/2* SNV/InDels	**15**	**12**	**3**	**-**	**-**
*HBA2*:c.427T>C/+	7	7	**-**	**-**	**-**
*HBA2*:c.369C>G/+	1	1	**-**	**-**	**-**
*HBA2*:c.377T>C/+	4	4	**-**	**-**	**-**
*HBA2*:c.-15C>G,c.369C>G/+	1	0	1	*HBA2*:c.369C>G/+	*HBA2*:c.-15C>G,c.369C>G/+
*HBA1*:c.84G>T/+	1	0	1	ND	*HBA1*:c.84G>T/+
*HBA2*:c.300 + 43G>A/+	1	0	1	ND	*HBA2*:c.300 + 43G>A/+
*HBA1*/2 SVs	**1**	**0**	**1**	**-**	**-**
--^SEA^/ααα^anti3.7^	1	0	1	--^SEA^/αα	--^SEA^/ααα^anti3.7^
*HBA1*/2 deletions & *HBA1*/2 SNV/InDels	**2**	**1**	**1**	**-**	**-**
-α^3.7^/*HBA2*:c.-15C>G,c.369C>G	1	0	1	-α^3.7^/*HBA2*:c.369C>G	-α^3.7^/*HBA2*:c.-15C>G,c.369C>G
-α^4.2^/*HBA2*:c.369C>G	1	1	-	-	-
*HBA1*/2 & *HBB* variants	**17**	**10**	**7**	-	**-**
--^SEA^/-α^3.7^ & *HBB*:c.52A>T/+	1	1	-	-	-
-α^3.7^/αα & *HBB*:c.315 + 180T>C/+	1	0	1	-α^3.7^/αα	-α^3.7^/αα & *HBB*:c.315 + 180T>C/+
-α^3.7^/αα & *HBB*:c.-100G>A/+	1	0	1	-α^3.7^/αα	-α^3.7^/αα & *HBB*:c.-100G>A/+
-α^3.7^/αα & *HBB*:c.316–179A>C/+	1	0	1	-α^3.7^/αα	-α^3.7^/αα & *HBB*:c.316–179A>C/+
-α^3.7^/αα & *HBB*:c.316–45G>C/+	1	0	1	-α^3.7^/αα	-α^3.7^/αα & *HBB*:c.316–45G>C/+
-α^4.2^/αα & *HBB*:c.-79A>G/+	1	1	-	-	-
-α^4.2^/αα & *HBB*:c.126_129delCTTT/+	2	2	-	-	-
-α^4.2^/αα & *HBB*:c.316–45G>C/+	1	0	1	-α^4.2^/αα	-α^4.2^/αα & *HBB*:c.316–45G>C/+
--^SEA^/αα & *HBB*:c.52A>T/c.-100G>A	1	0	1	---^SEA^/αα & *HBB*:c.52A>T/+	--^SEA^/αα & *HBB*:c.52A>T/c.-100G>A
--^SEA^/αα & *HBB*:c.126_129delCTTT/+	3	3	-	-	-
--^SEA^/αα & *HBB*:c.216_217insA/+	1	1	-	-	-
--^SEA^/αα & *HBB*:c.316–45G>C/+	1	0	1	--^SEA^/αα	--^SEA^/αα & *HBB*:c.316–45G>C/+
*HBA2*:c.369C>G/+ & *HBB*:c.126_129delCTTT/+	1	1	-	-	-
*HBA2*:c.427T>C/+ & *HBB*:c.216_217insA/+	1	1	-	-	-
*HBB* SNV/InDels	**27**	**25**	**2**	**-**	​
*HBB*:c.126_129delCTTT/+	13	13	​	-	-
*HBB*:c.316–179A>C/+	1	0	1	ND	*HBB*:c.316–179A>C/+
*HBB*:c.316–197C>T/+	4	4	-	-	-
*HBB*:c.-78A>G/+	4	4	-	-	-
*HBB*:c.52A>T/+	2	2	-	-	-
*HBB*:c.126_129delCTTT/c.79G>A	1	1	-	-	-
*HBB*:c.316–197C>T/c.52A>T	1	1	-	-	-
*HBB*:c.316–179A>C/c.52A>T	1	0	1	*HBB*:c.52A>T/+	*HBB*:c.316–179A>C/c.52A>T
Total	**149**	**135**	14	​	​

TGS, third-generation sequencing; SNV, single-nucleotide variant; InDels, insertion and deletions; SEA, south east asian; ND, not detected. Bold values indicate the thalassemia variant types identified in this study.

## Discussion

Leveraging TGS, this study presented the first comprehensive characterization of thalassemia epidemiology and genomic landscape in the neonatal population of Yulin region, a region endemic for thalassemia in southern China. Moreover, we systematically evaluated the effectiveness of TGS against conventional screening strategies from both strategic and technological perspectives, thereby informing evidence-based strategy for precision prevention and control of thalassemia in this region.

Yulin Region has implemented multiple thalassemia prevention and control initiatives. However, prior efforts have predominantly emphasized preconception carrier screening and prenatal diagnosis, while neonatal thalassemia screening remains undeveloped. To address this gap, we conducted thalassemia screening for 800 newborns in Yulin region by directly using TGS. The observed thalassemia carrier rate is higher than the previously documented rate for Yulin Region (He et al., 2018), indicating a serious public health concern in this region. This discrepancy is mainly attributable to two aspects: (1) population composition. This study exclusively enrolled neonates, whereas prior reports predominantly involved adult cohorts; and (2) screening methodology. Earlier studies employed sequential hematological-genetic workflows, whereas our approach utilized one-step method by directly using TGS. The most frequent genotypes of α-thalassemia and β-thalassemia in this subpopulation were--^SEA^/αα and *HBB*:c.126_129delCTTT heterozygotes, respectively, consistent with epidemiological data from Guangxi and Guangdong provinces but divergent from those reported in Yunnan and Hainan (Chen et al., 2022; Yu et al., 2022; Shang et al., 2017), These regional differences underscore the strong geographical stratification of thalassemia prevalence and genotype distribution across southern China.

Current thalassemia screening in China primarily employs hematological testing for initial screening, followed by confirmatory genetic analysis to identify the genotypes (Writing Group For Practice Guidelines For Diagnosis and Treatment Of Genetic Disease Medical Genetics Branch Of Chinese Medical Association, 2020a; Writing Group For Practice Guidelines For Diagnosis and Treatment Of Genetic Disease Medical Genetics Branch Of Chinese Medical Association, 2020b). However, these sequential strategies still have several limitations. First, according to the sequential strategies, only cases with positive hematological results are recommended for subsequent genetic analysis. Nevertheless, hematological tests are prone to false negatives for thalassemia silent variants and α-triplication. Second, conventional genetic assays including Gap-PCR and PCR-RDB target only a limited panel of common variants accounting for more than 95% of the Chinese population, posing risk of misdiagnosis and missed diagnosis of rare and complex structural variants (Shang et al., 2017; Zhang et al., 2019). Therefore, the conventional screening strategies fall short of supporting precision public heath interventions, especially in areas with high-prevalence, such as Yulin Region. In the past years, accumulating evidence has established TGS technology as a powerful tool for thalassemia detection (Luo et al., 2022a; Luo et al., 2022b). A prospective multi-center study confirmed 100% concordance between TGS and conventional methods without false negatives or false positives, and uniquely identified 34 additional variants all independently confirmed by designed PCR and Sanger sequencing, thereby confirming its clinical utility for identifying both α- and β-thalassemia genetic carrier status (Liang et al., 2021). Similarly, TGS also achieved superior accuracy in 278 fetuses from at-risk pregnancies. In contrast, PCR-based methods had one false positive and two false negatives (Liang et al., 2023). In this study, we conducted a systematic comparative evaluation of TGS against conventional sequential strategies in neonatal thalassemia screening. Conventional screening workflows only identified 146 carriers among 800 newborns with a rate of 18.25%, whereas TGS detected 195 carriers with a rate of 24.38%, representing an increment of 33.56% over conventional strategies. This increment arises from two main factors. First, TGS was applied to all participants, whereas conventional strategies restrict molecular testing exclusively to individuals with positive hematological results. As shown in Table 4, TGS identified globin gene variants in 7.57% of the participants with negative hematological results, including thalassemia silent and α-triplication. Critically, the detection of these variants also has clinical significance. For example, co-inheritance of α-triplication with β-thalassemia allele exacerbates α/β-globin chain imbalance, potentially leading to intermediate β-thalassemia (Lai et al., 2006). Second, TGS technology offers broader genomic coverage than conventional PCR-based genetic assays. In this cohort, TGS detected over 10 distinct rare variants, constituting a substantial proportion of thalassemia variants, especially in β-thalassemia variants, and enabled comprehensive, single-assay detection of all variant types. Whereas, at least two separate PCR-based assays are required to cover deletion and nondeletion variants. Moreover, TGS can directly determine the *cis/trans* configuration of multiple variants, eliminating the need for labor-intensive parental segregation analysis and significantly accelerating confirmatory reporting. With the cost for library preparation and sequencing reagent now reduces to approximately 20$ per sample, TGS is increasingly viable as a first-tier, population-scale neonatal screening platform, especially in areas with high-prevalence of thalassemia.

This study still has some limitations. First, the cohort only includes 800 consecutive neonates in Yulin Region, limiting generalizability and statistical power for precise regional prevalence estimation. Future expansion to larger, geographically stratified cohorts will refine epidemiological estimates and enhance characterization of the local thalassemia genomic landscape. Second, a subset of detected variants were classified as variants of uncertain significance (VUS), which cannot be used for clinical diagnosis or management decisions and required careful interpretation during genetic counselling. Third, the study exclusively offered conventional genetic testing to participants with positive hematological results in accordance with guidelines in clinical practice, whereas TGS to all participants, which may introduce verification bias. Besides, diagnostic accuracy metrics could not be robustly estimated due to partial orthogonal validation design. Finally, because newborns typically do not develop clinical manifestations until approximately 6 months after birth even in the most severe postnatal thalassemia cases, long-term follow-up analysis is warranted to predict genotype-phenotype correlation and validate the clinical predictive value of TGS findings. Such prospective data will be critical to establishing evidence-based genetic counseling during carrier screening and prenatal diagnosis, and defining the actual clinical utility of TGS in early-life thalassemia screening.

## Conclusion

This study represents the first application of TGS for population-based neonatal thalassemia screening in Yulin Region. It establishes a foundational epidemiological and genomic resource for thalassemia in this population, underscoring the urgent need for scalable, accurate neonatal screening to inform timely clinical management and preventive health care. Our work further provides evidence suggesting that TGS may serve as a viable option for neonatal thalassemia screening especially in endemic regions where early identification maximizes clinical and public health impact, thereby facilitating the implementation of precision prevention and control strategies.

## Data Availability

The original contributions presented in the study are included in the article/Supplementary Material, further inquiries can be directed to the corresponding author.
