# Supplementary material for: The value of third-generation sequencing for neonatal screening of thalassemia in the Yulin region of Southern China
Source: Front Genet. 2026 Jun 26;17:1843092. doi: 10.3389/fgene.2026.1843092 (PMC13350062; doi:10.3389/fgene.2026.1843092)
Supplement: Supplementary file 1 [file Table1.docx]

**Supplementary Table 1 Hemoglobin electrophoresis results of cases with rare variants**

| **Genotypes** | **Cases (n)** | **Age (days)** | **HbA (%)** | **HbA2 (%)** | **HbF (%)** | **Hb Bart’s(%)** |
| --- | --- | --- | --- | --- | --- | --- |
| ααα^anti3.7^/αα | 4 | 2.50±1.29 | 18.60±7.19 | 0.23±0.45 | 81.18±7.62 | 0 |
| --^SEA^/ααα^anti3.7^ | 1 | 1 | 24.50 | 0 | 74.80 | 0.70 |
| ααα^anti4.2^/αα | 1 | 1 | 15.50 | 0 | 84.50 | 0 |
| αααα^anti3.7^/αα | 1 | 1 | 11.50 | 0 | 88.50 | 0 |
| HBA2:c.-15C>G,c.369C>G/+ | 2 | 1.00±0.00 | 17.35±0.49 | 0 | 82.60±0.42 | 0.05±0.07 |
| -α^3.7^/HBA2:c.-15C>G,c.369C>G | 1 | 1 | 16.50 | 0 | 82.50 | 1.00 |
| HBA1:c.84G>T/+ | 2 | 1.50±0.71 | 20.15±4.74 | 0 | 79.80±4.67 | 0.05±0.07 |
| HBA2:c.300+43G>A/+ | 1 | 1 | 9.50 | 0 | 90.50 | 0 |
| HBB:c.316-179A>C/+ | 8 | 2.25±1.91 | 16.25±6.62 | 0 | 83.75±6.62 | 0 |
| -α^3.7^/αα & HBB:c.316-179A>C/+ | 1 | 1 | 15.20 | 0 | 84.50 | 0.30 |
| HBB:c.316-179A>C/c.-79A>G | 1 | 1 | 18.80 | 0 | 81.20 | 0 |
| HBB:c.316-179A>C/c.52A>T | 1 | 1 | 9.20 | 0.10 | 90.70 | 0 |
| HBB:c.315+180T>C/+ | 4 | 3.25±2.06 | 19.50±8.47 | 0 | 80.50±8.47 | 0 |
| -α^3.7^/αα & HBB:c.315+180T>C/+ | 1 | 4 | 19.50 | 0 | 79.90 | 0.60 |
| HBB:c.316-45G>C/+ | 3 | 3.00±1.73 | 16.17±3.07 | 0 | 83.83±3.07 | 0 |
| --^SEA^/αα & HBB:c.316-45G>C/+ | 1 | 1 | 26.10 | 0 | 71.60 | 2.30 |
| -α^3.7^/αα & HBB:c.316-45G>C/+ | 1 | 4 | 11.80 | 0 | 88.00 | 0.20 |
| -α^4.2^/αα & HBB:c.316-45G>C/+ | 1 | 1 | 31.50 | 0 | 68.20 | 0.30 |
| --^SEA^/αα & HBB:c.52A>T/c.-100G>A | 1 | 1 | 14.60 | 0 | 83.80 | 1.60 |
| HBB:c.-100G>A/+ | 1 | 1 | 20.90 | 0 | 79.10 | 0 |
| -α^3.7^/αα & HBB:c.-100G>A/+ | 1 | 1 | 11.90 | 0 | 88.00 | 0.10 |
| HBB:c.315+308delA/+ | 1 | 5 | 10.10 | 0 | 89.90 | 0 |
| HBB:c.315+5G>C/+ | 1 | 1 | 10.40 | 0 | 89.60 | 0 |
| **Total** | **40** | **-** | **-** | **-** | **-** | **-** |
